# Knockdown of Long Non-Coding RNA UCA1 Increases the Tamoxifen Sensitivity of Breast Cancer Cells through Inhibition of Wnt/β-Catenin Pathway

**DOI:** 10.1371/journal.pone.0168406

**Published:** 2016-12-15

**Authors:** Hongying Liu, Gang Wang, Lili Yang, Jianjun Qu, Zhihui Yang, Xiangyu Zhou

**Affiliations:** 1 Department of Genetics, Weifang Medical University, Weifang, Shandong, China; 2 Department of Surgical Oncology, Weifang People’s Hospital, Weifang, Shandong, China; 3 Department of Pathology, SouthWest Medical University of China, Luzhou, Sichuan, China; 4 Department of Vascular and Thyroid Surgery, the Affiliated Hospital of South Medical University of China, Luzhou, Sichuan, China; University of South Alabama, UNITED STATES

## Abstract

Acquired resistance to tamoxifen remains a major obstacle in breast cancer (BC) treatment, since the underlying mechanism has not been fully elucidated. The long non-coding RNA (lncRNA) urothelial carcinoma-associated 1 (UCA1) has been recently shown to be dysregulated and plays important roles in progression of breast cancer. In the present study, we aimed to investigate the biological role and clinical significance of UCA1 in BC drug resistance. Hence, we used quantitative PCR assay to evaluate the UCA1 expression in tissues from patients with BC as well as established tamoxifen-resistant BC cell lines *in vitro*. We tested the viability, invasive ability and apoptosis rate in MCF-7 and T47D cells using MTT assay, transwell assay and flow cytometry assay, respectively. The influence of UCA1 on tumorigenesis was monitored by *in vivo* mice xenograft model. The activation of Wnt/β-catenin signaling pathway was evaluated by immunofluorescence assay, western blot assay and luciferase reporter assay, respectively. We found that the expression of UCA1 positively correlated with the pathological grade and mortality of breast cancer patients, moreover, expressions of UCA1 was increased significantly in the tamoxifen-resistant cell lines compared with the wild type parental cells. Ectopic expression of UCA1 promoted cell survival and resistance to tamoxifen treatment, whereas inhibition of UCA1 enhanced tamoxifen sensitivity of BC cells and induced more apoptotic cells. In addition, tamoxifen-resistant cells exhibited increased Wnt signaling activation as measured by the TOP/FOP Wnt luciferase reporter assay and β-catenin protein level compared with parental MCF-7 and T47D cells, respectively. In line with these data, UCA1 depletion attenuated the activity of Wnt/β-catenin pathway activation and the tumorigenicity of the tamoxifen-resistant BC cells. Taken together, our data highlights the pivotal role of UCA1-Wnt/β-catenin signaling pathway in the tamoxifen resistance in breast cancer, which could be targeted to improve the effectiveness and efficacy of tamoxifen treatment in breast cancer.

## Introduction

Breast cancer is the most common female malignancy in the world and about 70% of them are estrogen receptor positive (ER^+^) [1]. Tamoxifen, an estrogen antagonist in the breast, is one of the standard hormone therapy for ER^+^ breast cancer in clinic. Although most patients benefit from this therapy, many tumors eventually recur because of the tamoxifen resistance [2, 3]. During the past decades, intensive efforts have been made to overcome the acquired drug resistance leading to the identification of complex factors/pathways contributing to tamoxifen resistance including the growth factor receptor networks (EGFR/HER2), the NF-κB pathway as well as the contribution of cancer stem cells [1, 4, 5]. However, tamoxifen resistance still remains a major obstacle in clinical practice. Thus, further insights into the mechanisms underlying acquired tamoxifen resistance will help to improve the effectiveness and efficacy of ER^+^ breast cancer treatment with tamoxifen.

Long non-coding RNA (LncRNA) is defined as a class of non-protein coding transcripts over 200 nucleotides [6]. Emerging evidences have indicated that lncRNAs play critical roles in the cancer development by regulating the proliferation and differentiation, apoptosis, and cell cycle of cancer cells [7]. They have also been shown to contribute to the chemoresistance of various cancers [8]. The lncRNA urothelial carcinoma associated 1 (UCA1) was originally identified as a urine marker encoding 1439 bp transcript in bladder cancer [9]. Increasing evidences have shown that UCA1 is dysregulated in other cancers, such as bladder carcinoma, colorectal, melanoma, breast, gastric, and esophageal squamous cell carcinoma [10]. Recent studies also demonstrated that the expression of UCA1 was increased in the breast cancer [11], which promoted the growth of breast cancer by suppressing the tumor suppressor p27 [12], highlighting the important roles of UCA1 in breast cancer development. However, whether UCA1 plays any roles in the acquired tamoxifen resistance in breast cancer is not reported so far.

## Materials and Methods

### Patients selection

This was a case-control pilot study developed at Weifang Medical University with the analysis of 54 hormone receptor positive (HR+) breast cancer patients treated at Department of Surgical Oncology, 14 non-tumor donors were used as the normal control. 30 primary tumor specimens (stage I & stage II) and 24 advanced tumor specimens (Stage III & Stage IV) with breast cancer were selected (aging from 34–76 with median age 53), and all samples were collected pre-tamoxifen therapy. Exclusion criteria were bilateral disease and pregnancy concomitant with the diagnosis of breast cancer. The tumor samples were obtained in accordance with protocols approved by the Institutional Ethics Committee at the Weifang Medical University, and the written informed consent was obtained by all patients included in this study. This study was approved by the Institutional Ethics Committee at the Weifang Medical University.

### Cell culture and transfection

MCF-7 and T47D cell lines were purchased from American Type Culture Collection (ATCC, Manassas, VA, USA) and maintained in Dulbecco’s modified Eagle’s medium (DMED) supplemented with 10% FBS, 2 mM glutamine, 100 U/ml penicillin and 100 μg/ml streptomycin. The tamoxifen resistant variant cells (MCF-7-R and T47D-R) were generated by continuously exposing to increasing doses of 4-hydroxytamoxifen (4-OHT; Sigma-Aldrich, Shanghai, China) up to 10 μM and 6μM, respectively. All cells were maintained at 37°C in a humidified atmosphere containing 5% CO_2_. For the overexpression or knockdown of UCA1, lentiviral vectors harboring UCA1 expressing plasmid were infected, the small interfering RNA targeting UCA1 (si-UCA) and the scramble non-target control were transfected into the cells reaching the exponential growth phase using HiPerFect transfection reagent (Qiagen, Valencia, CA, USA) according to manufacturer’s instructions, respectively. After 12 hours, the medium was replaced with fresh complete medium, and cells were cultured for another 2–3 days before further experiments.

### RT-PCR

The total RNA was extracted from cells using Trizol reagent (Invitrogen, Carlsbad, CA, USA). 1 μg of total RNA was treated with DNase I (Invitrogen, Carlsbad, CA, USA) and the cDNA was synthesized in vitro using SuperScript^®^ III First-Strand Synthesis Kit (Invitrogen, Carlsbad, CA, USA). The mRNA level was determined using 7900HT (Applied Biosystems, Foster City, CA, USA) real time PCR system using SYBR^®^ Green Master Mix (Applied Biosystems, Foster City, CA, USA). Glyceraldehyde-3-phosphate dehydrogenase (GAPDH) was used as an internal control, and the relative expression were calculated using the 2^-ΔΔCt^ method for at least three independent experiments. The sequences of primers sued in this study are as follows: *UCA1* F: 5'-TTTGCCAGCCTCAGCTTAAT-3', R: 5'-TTGTCCCCATTTTCCATCAT-3'; *GAPDH* F: 5'-CCACCCATGGCAAATTCCATGGCA-3', R: 5'-TCTAGACGGCAGGTCAGGTCCACC-3'.

### Cell proliferation, apoptosis, and migration assay

After treatments as designed, cells were dissociated from the culture wells with 0.25% trypsin and counted using Bio-Rad cell counter (Bio-Rad Laboratories, Hercules, CA, USA). To explore the effect of tamoxifen on cell survival, MTT assay was performed according to manufacturer’s instructions (Amylet Scientific, Wuhan, Hubei, China). Briefly, 2000 cells/well were plated into a 96-well flat bottomed plate, allow cells to grow with or without tamoxifen treatment, incubate cells to assigned time point and then add MTT reagent into the media for a few minutes then read plates at wavelength 540nm. The relative proliferation rate was normalized by the day 0 group, and summarized for three independent experiments.

Apoptosis of cells was determined by dual staining with FITC-Annexin V (BD, Franklin Lakes, NJ, USA) and propidium iodide (PI, Sigma-Aldrich, Shanghai, China). In brief, scramble and si-UCA1 transfected cells were seeded onto culture plate and incubate with different concentration of tamoxifen (0~100 nM) for 24 hrs. Cells were dissociated using 0.25% trypsin and centrifuged at 1200 rpm for 5 min. Pellet was resuspended in binding buffer and stained with FITC-Annexin V and PI. After staining, cells were analyzed with the FACS Calibur system (BD, Franklin Lakes, NJ, USA).

Cell migration assay was carried out using transwell culture system. A total of 5×10^4^ cells were plated into the upper chamber of the transwell chamber. The lower chamber was filled with culture medium. 24 hrs later, the non-migrated cells on the upper side of the membrane were removed by cotton swabs. The migrating cells on the lower side of the insert filter were fixed with formaldehyde for 10 min and stained the cells with 1% crystal violet solution for 20 min. The number of cells was counted and imaged using the light microscope (Olympus, Tokyo, Japan).

### Tumorigenesis assay

The animal use and care were carried out in accordance with the guidelines by the U.S. National Institute of Health Guide for the Care and Use of Laboratory Animals. The protocol was approved by the animal care and ethics committee of Weifang Medical University. Scramble or -UCA1 infected MCF-7-R and T47D-R cells using lentivirus (about 5×10^6^) were mixed with matrigel, each mouse (10–12 w.o.) received a subcutaneous injection in the flank of the hind leg in a volume of 100 μl of matrigel-cell suspension. Tamoxifen (3 mg/kg) was applied i.p. to each mouse for 24 days. Tumors were measured every 3 days, and the length (L) and width (W) of each tumor was measured with calipers and the volume was calculated using the equation: V = (L×W^2^) ×0.5 for 24 days after the injection of cancer cells. Mice were sacrificed after 24 days using cervical dislocation following CO_2_ inhalation.

### Immunohistochemical staining and scoring

Cultured cells or tissue were fixed with 4% paraformaldehyde for 20 min and washed with PBS three times followed by blocking with 10% normal goat serum plus 1% BSA (Sigma-Aldrich, Shanghai, China) for 45 min, and incubated with rabbit anti-β-catenin (1:100) (Santa Cruz Biotechnology, Dallas, TX, USA) at 4°C in the dark overnight. After washing three times with PBS, cells and tissue were further incubated with cy3-conjugated goat anti-rabbit and HRP-conjugated goat anti-rabbit secondary antibodies for 1 h at 37°C. Subsequently, the slides were cover slipped with mounting medium (Dako, Dalian, Liaoning, China) containing DAPI to counter stain the nuclei and imaged with fluorescence or bright field microscope. Scoring for the slides were performed according to a previous publication [13]. Briefly, β-catenin staining with a nuclear localization pattern was examined by two independent pathologists with bright field microscope, and any nuclear staining of breast cancer cells was assumed as positive. Intensity of staining was scored on a scale of 0–3+ as follows: no specific staining (0), weak (1+), moderate (2+), and strong (3+). The Pearson correlation analysis was used to establish the relationship between UCA1 gene expression and nuclear β-catenin levels.

### Western blot

The protein expression levels of β-catenin and β-actin were analyzed with western blot assay. After treatments, cells were harvested in RIPA lysis buffer (Beyotime Institute of Biotechnology, Shanghai, China) supplemented with protease inhibitor cocktail (Sigma-Aldrich, Shanghai, China). Equal amount of proteins was resolved in SDS-PAGE and transferred to polyvinylidene fluoride (PVDF) membrane. Membranes were blocked with 5% non-fat dry milk in TBST buffer and incubated with primary antibodies at 4°C overnight. After washing 3 times with TBST buffer, the membranes were incubated HRP-conjugated secondary antibodies for 1 hr at room temperature. Then the membranes were washed, developed using chemiluminescence substrate, and imaged. The antibodies used were: rabbit anti-β-catenin (Abcam, Shanghai, China), mouse anti-β-actin (Abcam, Shanghai, China), anti-mouse HRP (Sigma-Aldrich, Shanghai, China) and anti-rabbit HRP (Sigma-Aldrich, Shanghai, China).

### Statistics

Data are presented as Mean ± SD. of at least three independent experiments except other statement. Significance of means between two groups is determined by student’s *t*-test. Difference at different time points among groups was evaluated by repeated measures analysis of variance (ONE-way ANOVA). Correlation analysis for the nuclear β-catenin level and the expression of lncRNA UCA1 in human breast cancer tissues was evaluated with Spearman's Rank-Order analysis. A *p*-value less than 0.05 was considered significantly different.

## Results

### Correlation between the lncRNA UCA1 expression and breast cancer development

**Fig 1:**
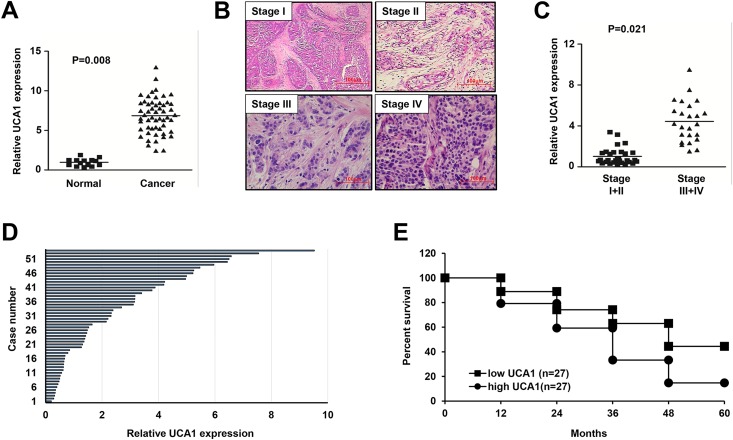
The lncRNA UCA1 expression correlates with development of breast cancer. (A) Analysis of the lncRNA UCA1 expression in normal and breast cancer tissues from healthy donor or patients using quantitative RT-PCR (qRT-PCR). Each sample was triplicated, GAPDH was used as the endogenous control, and the formula 2^-ΔΔ Ct^ was used to calculate the relative expression. (B) Representative H&E staining for the breast cancer tissues with different pathological stage I-IV. Magnification, 200×. (C) Analysis of the lncRNA UCA1 expression using qRT-PCR in two groups of breast cancer with different pathological stages (stage I+II vs. stage III+IV). (D) Expression pattern of the lncRNA UCA1 in all 54 cases of breast cancer samples. (E) Relationship between the survival probability of breast cancer patients and the lncRNA UCA1 expression level. 54 patients were divided into the UCA1 high and low groups by the median value, and the follow-up was done up to 5 years.

Firstly we detected the lncRNA UCA1 expression in all human breast cancer tissues enrolled in this study. In consistent with the previous study [11], we also detected significantly increased expression of lncRNA UCA1 in the breast cancer group compared with the normal control group (p<0.05) (Fig 1A). According to pathological grading, all 54 cases of breast cancer were classified as grade I, II, III and IV (Fig 1B), then were divided into two stage I+II group and stage III+IV group, and the expression level of lncRNA UCA1 was determined using quantitative RT-PCR (qRT-PCR). Our result demonstrated that the expression of lncRNA UCA1 was significantly higher in the stages III + IV group than that in the stage I + II group (p<0.05), indicating a positive correlation between the severity of breast cancer and the expression of UCA1 (Fig 1C). In line with this, when we divided all 54 patients into two groups according to the median value of lncRNA UCA1 (Fig 1C), we found that the survival rate dropped in breast cancer patients with high expression level of lncRNA UCA1 when compared with those with low expression of UCA1 (Fig 1D). These results suggest that lncRNA UCA1 contributes to the development of breast cancer.

### Manipulation of LncRNA UCA1 increases cell viability of breast cancer cells to tamoxifen treatment

**Fig 2:**
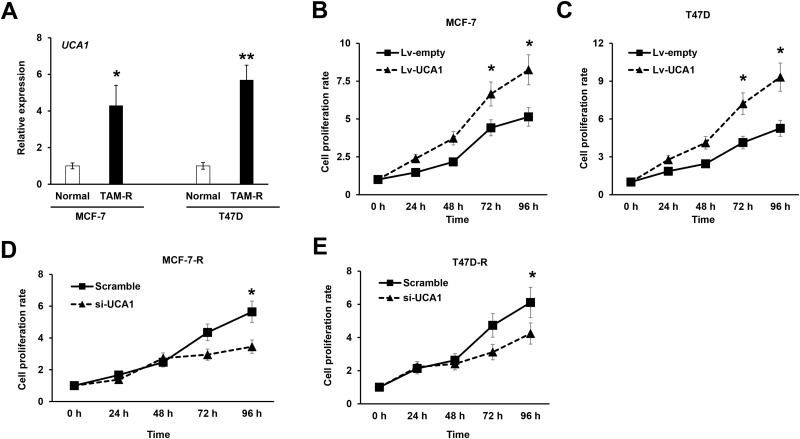
Manipulation of LncRNA UCA1 expression changes breast cancer cell drug resistance. (A) qRT-PCR analysis shows the lncRNA UCA1 expression in the tamoxifen-resistant and their parental breast cancer cells. Normal, the parental MCF-7 or T47D cells; TAM-R, tamoxifen resistant MCF-7 or T47D cells. Overexpression of lncRNA UCA1 using lentivirus (Lv-empty vector or Lv-UCA1) in MCF-7 cells (B) or T47D cells (C) promotes the proliferation of breast cancer cells detected with MTT assay. Knockdown of lncRNA UCA1 using small interfering RNA targeting UCA1 (si-UCA1) decreases the proliferation of tamoxifen-resistant MCF-7 (D) or T47D (E) breast cancer cells, respectively. All data represents at least three independent experiments. *, *P*<0.05; **, *P*<0.01.

To study the contribution of lncRNA UCA1 to tamoxifen resistance of breast cancer, we firstly established two tamoxifen-resistant breast cancer cell lines from the parental MCF-7 and T47D cells, and examined the expression of lncRNA UCA1 in these tamoxifen-resistant breast cancer cells (MCF-7-R and T47D-R, respectively). As shown in the Fig 2A, the expression of lncRNA UCA1 was dramatically increased in the tamoxifen-resistant MCF-7 and T47D cells compared with their parental cells, suggesting that lncRNA UCA1 may play important roles in the tamoxifen resistance of breast cancer (Fig 2A). To further confirm the contribution of lncRNA UCA1 to tamoxifen resistance, we overexpressed lncRNA UCA1 expression in both MCF-7 and T47D cells using lentivirus, and we found that the proliferation rate was significantly increased in both cell lines (Fig 2B and 2C). Consistently, knockdown of lncRNA UCA1 in the tamoxifen-resistant MCF-7-R and T47D-R cells attenuated the proliferation of both cell lines (Fig 2D and 2E). Taken together, these data indicated that lncRNA UCA1 contributes to the resistance of breast cancer cells to tamoxifen.

### Knockdown of lncRNA UCA1 enhances breast cancer chemo-sensitivity to tamoxifen

**Fig 3:**
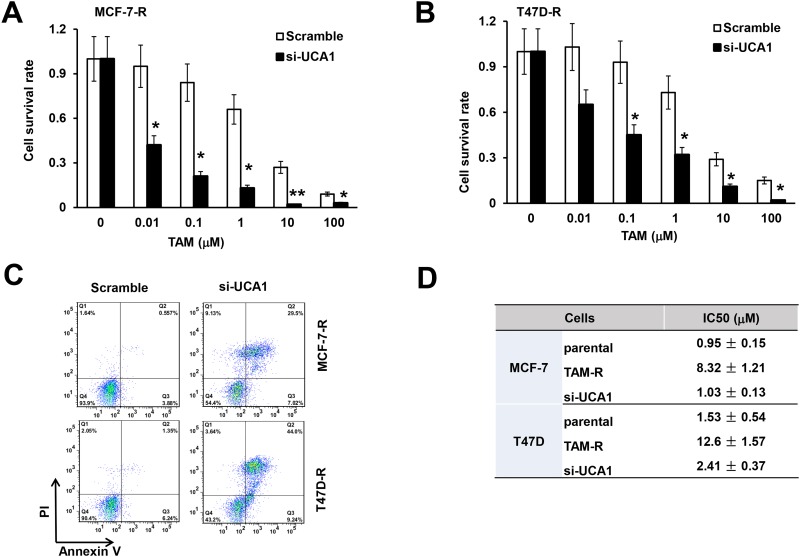
Knockdown of lncRNA UCA1 enhances breast cancer chemo-sensitivity. Knockdown of UCA1 decreased cell survival of MCF-7-R (A) and T47D-R (B) cells from tamoxifen treatment. (C) Representative flow cytometry showed the apoptosis ofMCF-7-R and T47D-R cells to tamoxifen treatment after transfected with scramble control or si-UCA1 using FITC-Annexin V and PI staining. (D) Summary of the calculated half maximal inhibitory concentration (IC50) of the wild type (parental), tamoxifen resistant (TAM-R), and lncRNA UCA1 knockdown (si-UCA1) breast cancer cells. All data represents at least three independent experiments. *, *P*<0.05; **, *P*<0.01.

To examine the effect of lncRNA UCA1 on tamoxifen sensitivity of breast cancer cells, we compared the survival rate of breast cancer cells transfected with scramble control or small interfering RNA targeting lncRNA UCA1. As shown, survival rate of MCF-7-R and T47D-R cells decreased gradually with increasing concentrations of tamoxifen (Fig 3A and 3B, blank bar chats). More importantly, when the MCF-7-R and T47D-R cells were transfected with siUCA1 rather than the scramble control, cell viability at each concentration of tamoxifen decreased significantly (Fig 3A and 3B, black bar chats). Flow cytometry analysis revealed that after the knockdown of lncRNA UCA1, the apoptotic of MCF-7-R and T47D-R cells was greatly enhanced upon tamoxifen treatment (Fig 3C), which means lncRNA UCA1 would protect breast cancer cells from tamoxifen induced apoptosis. After calculation, although the half maximal inhibitory concentration (IC50) increased almost 10 folds in the TAM-R cells, knockdown of lncRNA UCA1 backtracked the IC50 of TAM-R cell to almost the parental level (Fig 3D). These data indicate that knockdown of lncRNA UCA1 increased the tamoxifen sensitivity of breast cancer cells and promoted apoptosis.

### LncRNA UCA1 regulates the migration of breast cancer cells

**Fig 4:**
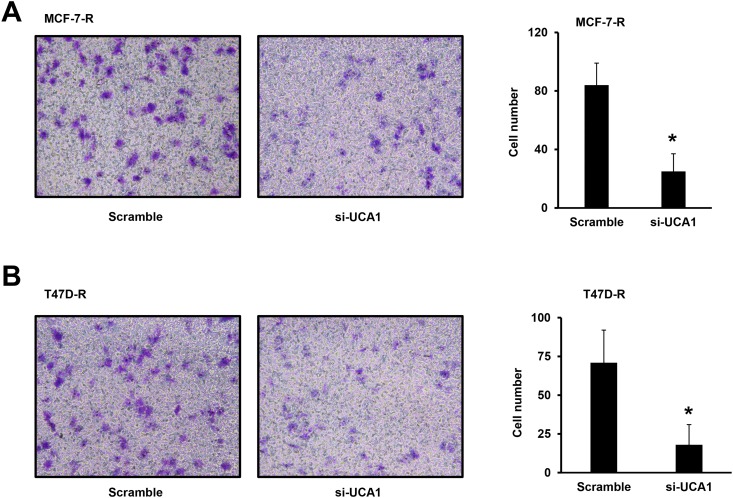
Knockdown of lncRNA UCA1 obstructs the migration of breast cancer cells. Representative pictures shows numbers of migrated of MCF-7-R (A) or T47D-R (B) breast cancer cells Transfected with scramble control or si-UCA1. The following histogram summarized cell numbers of 12 pictures from 3 independent experiments. *, *P*<0.05.

Next, we investigated the effect of lncRNA UCA1 on the migration ability of breast cancer cells using transwell assay. We compared the migration ability of TAM-R cells transfected with scramble or si-UCA1 as above descripted. As shown in the representative pictures, the number of MCF-7-R migrated to the lower chamber was significantly decreased after the transfection of si-UCA1 (Fig 4A). A similar phenotype was also observed in the T47D-R cells (Fig 4B). These data suggest that loss of function of lncRNA UCA1 inhibits the migration of breast cancer cells.

### Suppression of lncRNA UCA1 attenuates the tumorigenicity of tamoxifen-resistant breast cancer cells

**Fig 5:**
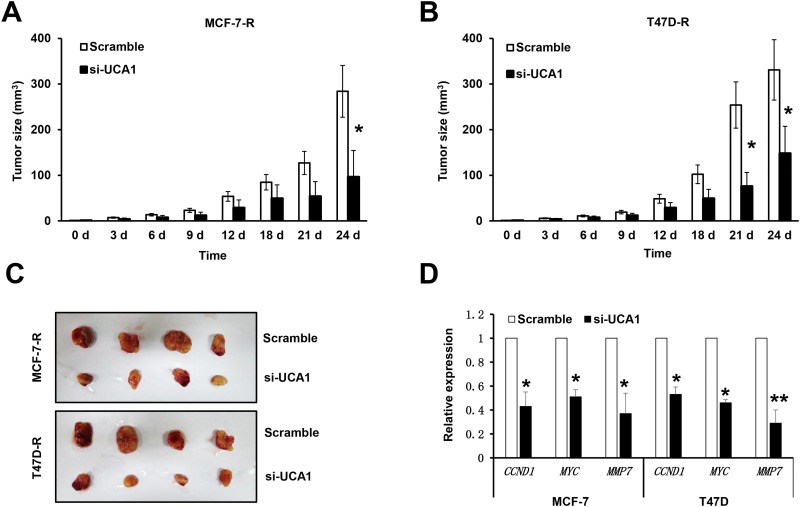
Inhibition of the tumorigenicity of tamoxifen-resistant breast cancer cells after knockdown of lncRNA UCA1. Bar graphs shows the development of tumors generated by MCF-7-R (A) and T47D-R (B) cells transfected with scramble (blank bars) or si-UCA1 (black bars). (C) Representative pictures shows the tumors generated by MCF-7-R and T47D-R cells transfected with scramble or si-UCA1 24 days after injection. Each group contained 5 cases and 4 mice were housed in each case. (D) qRT-PCR data showed the expressions of downstream target genes of the Wnt/β-catenin signaling in tumor tissues generated from MCF-7-R or T47D-R cells transfected with scramble (blank bar) or si-UCA1 (black bar). All data represents at least three independent experiments. *, *P*<0.05; **, *P*<0.01.

To confirm the above established effects of lncRNA UCA1 on breast cancer cells *in vivo*, we analyzed the effect of knockdown of lncRNA UCA1 on the tumorigenicity of MCF-7-R and T47D-R cells using the xenograft model in nude mice. After the tumor size was over 15mm, tamoxifen (3 mg/kg) was administered i.p. every 3 days for 24 days. As shown in Fig 5A and 5B, with the treatment of tamoxifen, the size of tumors generated from MCF-7-R or T47D-R cells transfected with scramble control were greatly suppressed at each time point than those cancer cells transfected with si-UCA1 (Fig 5A and 5B). At day 24, the tumors were taken out and processed for other experiments. The size of tumors was dramatically decreased by the knockdown of lncRNA UCA1 in MCF-7-R and T47D-R cells (Fig 5C). These results suggest that knockdown of lncRNA UCA1 enhanced the sensitivity of breast cancer to tamoxifen.

### LncRNA UCA1 inhibition prevents the nuclear translocation of β-catenin

**Fig 6:**
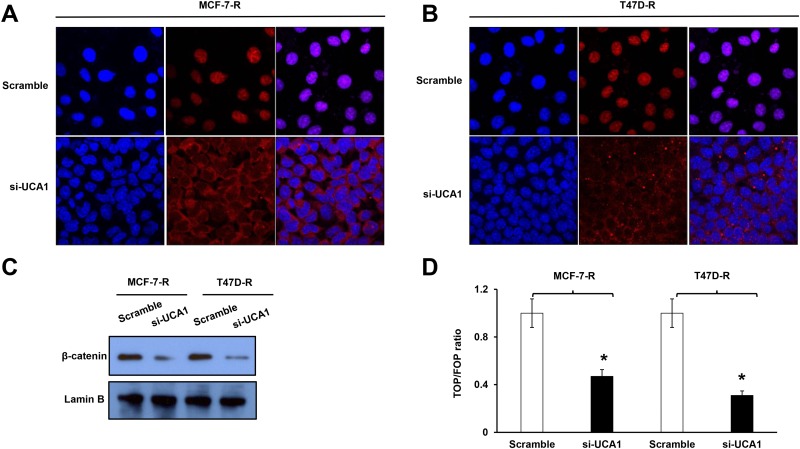
The lncRNAUCA1 promotes Wnt signaling activity by facilitating β-catenin cyto-nuclear translocation. Shown are immunocytochemistry staining for the localization of β-catenin in MCF-7-R (A) and T47D-R (B) cells transfected with scramble control or si-UCA1. Meganification, 400×. (C) Western blot assay shows the decreased β-catenin protein level in the nuclear fraction from cells transfected with si-UCA1. Nuclear Lamin B serves as the loading control. (D) TOP/FOP assay showing the decreased activity of Wnt signaling in the tamoxifen-resistant breast cancer cells when UCA1 was knockdown. *, *P*<0.05.

As previously revealed, Wnt/β-catenin pathway plays important roles in maintaining the stem-like features of breast cancer cells making they resistant to chemotherapy including tamoxifen [14]. To investigate the mechanism underlying the modulation of tamoxifen resistance by lncRNA UCA1, we examined the expression of β-catenin and the sub-cellular localization in tamoxifen-resistant cells with or without knockdown of lncRNA UCA1, since cyto-nuclear translocation is an important mechanism of Wnt signaling activation [15]. We used immunocytochemistry staining to examine the β-catenin level and localization. As shown in the immunocytochemistry results, β-catenin was mainly localized in the nuclear in tamoxifen-resistant MCF-7-R and T47D-R cells (Fig 6A and 6B). However, it was largely presented in the cytoplasm when lncRNA UCA1 was suppressed by si-UCA1 (Fig 6A and 6B). Furthermore, the cytoplasm and nuclear protein fractions were isolated, and Western blot assay was used to detect the nuclear level ofβ-catenin in the MCF-7-R and T47D-R cells transfected with scramble control or si-UCA1, respectively. Results showed that knockdown of lncRNA UCA1 significantly suppressed the nuclear β-catenin level in both cells (Fig 6C). In line with these data, the TOP/FOP reporter assay also demonstrated that the Wnt/β-catenin signaling was greatly inhibited by the lncRNA UCA1 knockdown (Fig 6D).

### Correlation of the expression of lncRNA UCA1 and the nuclear β-catenin protein level in vivo

**Fig 7:**
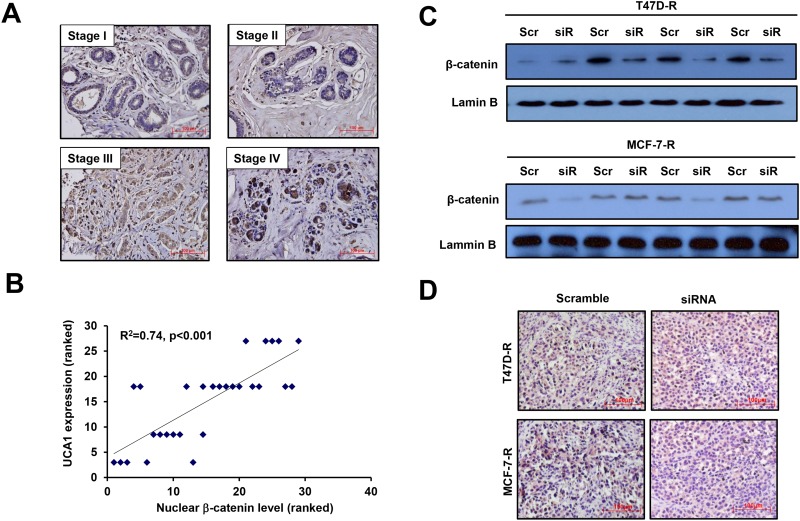
Correlation of the expression of lncRNA UCA1 and the nuclear β-catenin protein level *in vivo*. (A) Immunohistochemistry staining of human breast cancer tissue shows the nuclear different β-catenin level in the different pathological stage I-IV. (B) Spearman's Rank-Order Correlation analysis for the nuclear β-catenin level and the expression of lncRNA UCA1 in human breast cancer tissues. (C) Western blot shows the nuclear protein level of β-catenin in the tumors generated from MCF-7-R (upper panel) and T47D-R (lower panel) cells with UCA1 knocked down. Lamin B served as the loading control. (D) Immunohistochemistry staining shows the nuclear β-catenin level in tumor tissues generated from MCF-7-R and T47D-R cells, respectively. Magnification, 200×. Scale bar, 100μm.

Established the contribution of lncRNA UCA1 on Wnt/β-catenin signaling activity using in vitro model, we further examined the nuclear β-catenin level in different stages of breast cancer patients. In consistent with the above in vitro experiments, the immunohistochemical staining showed that the expression of β-catenin was higher in the nuclei of stage III and IV than that in stage I and II breast cancer patients (Fig 7A). We also used the Spearman's Rank-Order Correlation analysis to define the correlation between the nuclear β-catenin level and the expression of lncRNA UCA1 in human breast cancer tissues, and the results indicated a highly positive correlation (R^2^ = 0.77, p<0.001) (Fig 7B). Furthermore, tumor tissues were taken from the xenograft for further analysis. Western blot showed that suppressed expression of β-catenin was presented in the nuclear protein extractions from the tumors generated by si-UCA1-transfected cells compared with scrambled control in both MCF-7-R and T47D-R cells (Fig 7C). Immunohistochemistry staining also confirmed that nuclear β-catenin was obviously down-regulated in the tumors generated from si-UCA1 MCF-7 or T47D cells (Fig 7D). These data suggest that UCA1 promotes the nuclear translocation of β-catenin in patients and the *in vivo* model.

## Discussion

In recent years, there have been a great treatment advances against breast cancer leading to improved survival for people of all ages and races with all stages [16]. However, acquired tamoxifen resistance remains the major obstacle in breast cancer treatment. Emerging evidence has shown that lncRNAs play important roles in various aspects of cancer biology and contribute to the development of drug resistance in chemotherapy of cancers [8]. It has recently been shown that lncRNA UCA1 increases the chemoresistance of bladder cancer [17]. In consistent with the effect of lncRNA UCA1 in bladder cancer, we demonstrated in the current study that the expression of lncRNA UCA1 was elevated in late stages of breast cancer and positively correlates with the mortality of patients. In addition, upregulation of lncRNA UCA1 was also detected in the tamoxifen-resistant cancer cells compared with their parent cells. These data suggest that lncRNA UCA1 may play important roles in the development of tamoxifen resistance.

Tamoxifen resistance is a complex progress involving the inappropriate activation of ER-mediated epidermal growth factor receptor (EGFR) signaling pathway which promotes the proliferation and survival of cancer cells, rendering them cancer stem cell-like properties [1, 5, 18]. Our current study demonstrated that knockdown of lncRNA UCA1 decreased the cell survival and increased apoptosis of tamoxifen-resistant cells when exposed to tamoxifen, indicating that lncRNA UCA1 may function to suppress the apoptosis and promote the proliferation of breast cancer cells in tamoxifen treatment. In addition, knockdown of lncRNA UCA1 significantly attenuated the migration ability of tamoxifen-resistant cells. In line with the in vitro experiment, the tumorigenicity of tamoxifen-resistant cells was dramatically attenuated when lncRNA UCA1 was inhibited in the xenograft mouse model, indicating that lncRNA UCA1 contributes to the tamoxifen-resistance. Although, increase in expression of lncRNA UCA1 with worse grade of breast cancer may not have a definite association of lncRNA UCA1 with tamoxifen resistance, since all these samples were taken before treatment. However, drug resistant genes are often associated with cancer progress, metastasis and invasion [19–21]. In this study, we firstly found UCA1 expression level was correlated with ER+ breast cancer progress and migration, then we found knockdown of UCA1 could enhance chemosensitivity of breast cancer cell to tamoxifen, therefore these convincing evidences indicate a close correlation between the lncRNA UCA1 and the breast cancer drug resistance to tamoxifen.

Long-term treatment with tamoxifen leads to the redistribution of ER-α to extra-nuclear sites facilitating the interaction with EGFR (18). Emerging evidences have shown a close interaction between Wnt/β-catenin signaling and ER signaling [22, 23]. Beta-catenin has been shown to be associated with the redistribution of ER in the heart [24]. In addition, increased Wnt/β-catenin signaling has been shown to be responsible for maintaining the stem-like features of breast cancer cells making they resistant to chemotherapy including tamoxifen [14, 25]. In line with this, we demonstrated that inhibition of lncRNA UCA1 in the tamoxifen-resistant cells prevents the translocation of β-catenin to the nuclear and inhibits the activity of Wnt signaling. These results demonstrate that lncRNA UCA1 may increase the activity of Wnt/β-Catenin signaling via promoting the nuclear translocation of β-catenin, which contributes to the extranuclear redistribution of ER. Although, the current study did not investigate the mechanism of lncRNA UCA-1 affects β-catenin level and sub-cellular localization, some literatures have demonstrated that lncRNAs regulates Wnt signaling via regulating Frizzled, interacting with hnRNP-K, or recruiting the SWI/SNF complex to the TCF7 promoter [26–28], these clues together with our finding are valuable for further investigations to the topic.

In conclusion, we demonstrate that the knockdown of lncRNA UCA1 inhibits the Wnt/β-catenin signaling and increases the tamoxifen sensitivity by promoting the apoptosis of breast cancer cells when exposed to tamoxifen. These results suggest that the tamoxifen resistance could be overcome by targeting the lncRNA UCA1 or Wnt/β-catenin signaling pathway.
